# MiR-302a Regenerates Human Corneal Endothelial Cells against IFN-γ-Induced Cell Death

**DOI:** 10.3390/cells12010036

**Published:** 2022-12-22

**Authors:** Se-Hie Park, Jin-Sun Hwang, Sun-Hee Oh, Young-Joo Shin

**Affiliations:** 1 Department of Ophthalmology, Hallym University Medical Center, College of Medicine, Hallym University, Seoul 07441, Republic of Korea; 2 Hallym BioEyeTech Research Center, Hallym University College of Medicine, Seoul 07442, Republic of Korea

**Keywords:** human corneal endothelial cells, interferon-γ, miR-302a, senescence

## Abstract

Damage to human corneal endothelial cells (hCECs) leads to bullous keratopathy because these cells cannot be regenerated in vivo. In this study, we investigated the protective role of microRNA (miR)-302a against interferon-γ (IFN-γ)-induced senescence and cell death of hCECs. Cultured hCECs were transfected with miR-302a and treated with IFN-γ (20 ng/mL) to evaluate the protective effect of miR-302a on IFN-γ-induced cell death. Senescence was evaluated by the senescence-associated β-galactosidase (SA-β-gal) assay, and the secretion of senescence-associated secretory phenotype (SASP) factors was analyzed. Mitochondrial function and endoplasmic reticulum (ER) stress were assessed. We revealed that miR-302a enhanced the cell viability and proliferation of hCECs and that IFN-γ increased the cell size, the number of SA-β-gal-positive cells, and SASP factors, and arrested the cell cycle, which was eliminated by miR-302a. miR-302a ameliorated mitochondrial oxidative stress and ER stress levels which were induced by IFN-γ. IFN-γ decreased the mitochondrial membrane potential and promoted autophagy, which was eliminated by miR-302a. The in vivo study showed that regeneration of rat CECs was promoted in the miR-302a group by inhibiting IFN-γ and enhancing mitochondrial function. In conclusion, miR-302a eliminated IFN-γ-induced senescence and cellular damage by regulating the oxidative and ER stress, and promoting the proliferation of CECs. Therefore, miR-302a may be a therapeutic option to protect hCECs against IFN-γ-induced stress.

## 1. Introduction

The cornea is the transparent window of the eye that refracts light [1]. Corneal endothelial cells (CECs) are localized in the innermost part of the cornea, which pumps water out of the corneal stroma to keep the cornea transparent [2]. The reduction in the number of human corneal endothelial cells (hCECs) due to trauma, surgery, or genetic abnormalities results in an invisible and painful corneal edema, known as bullous keratopathy, because the hCECs do not regenerate in vivo [3]. There is no effective treatment for bullous keratopathy except for corneal transplantation [4]. Corneal transplantation has several limitations including rejection and gradual decrease in number of hCECs, which requires repeat transplantation [4]. Therefore, it is necessary to develop a novel method to induce regeneration of hCECs as the treatment of hCECs. Gene therapy using gene activation, such as SRY-box transcription factor 2 (SOX2) or SIRT1, has been suggested to regenerate the CECs. However, plasmid transfection leads only to continuous proliferation, which is undesirable, and proliferation should be halted in order to not cause cancer and to maintain the functions. Therefore, interest in microRNAs (miRNA) has arisen to suppress the signal that stops the proliferation of the corneal endothelium. 

miRNAs have been reported to bind to the target mRNAs and negatively regulate their expression [5]. Although thousands of human genes are miRNA targets, the function of individual miRNAs remains unknown [5]. Studying the function of individual miRNAs in hCECs is important to understand the pathology of CECs, and to develop the treatment for their regeneration. miR-302a is an embryonic stem cell (ESC)-specific miRNA that targets the serine-threonine kinase 1 (AKT1), cyclin-dependent kinase inhibitor (CDKN)-1A, CDKN1B, metastasis-associated in colon cancer-1, ras homolog family member C, transforming growth factor beta receptor 2 (TGFBR2), RAB5C, orthodenticle homeobox 2, GRB2-associated binding protein 2 (GAB2), and the extracellular signal-regulated kinases (ERKs) [6,7,8,9,10]. Stem cell markers, such as NANOG, octamer-binding transcription factor 4 (OCT4), and SOX2 control the expression of the miR-302/367 gene to control the stemness, self-renewal, cell proliferation, and survival of the stem cells [7,10,11]. 

Interferon-γ (IFN-γ), a pro-inflammatory cytokine, inhibits cell proliferation and activates inflammation and programmed cell death through the NF-κB signaling pathway [12,13]. Inhibiting the IFN-γ/NF-κB pathway is important for the preservation of cells. miR-302a inhibits the interferon regulatory factor-5 and associated cytokine secretion [14] through suppressing the nuclear translocation of NF-κB by inhibiting phosphorylation of IκB kinase complex β via targeting IRAK4 and the receptor activator of the NF-κB ligand [15,16]. The up-regulation of the inflammatory and immune responses in the corneal endothelium and Descemet’s membrane are shown in CEC diseases [17]. IFN-γ induces inflammasome-mediated CEC death [18]. CEC diseases are related to the NF-κB signaling pathway [19]. Therefore, in this study, we investigated the regulatory role of miR-302a in the regeneration and IFN-γ-induced cell death of hCECs.

## 2. Materials and Methods

### 2.1. Cell Culture and Transfection

This study was performed in accordance with the tenets of the Declaration of Helsinki, and was reviewed and approved by the Institutional Review Board/Ethics Committee of Hallym University Kangnam Sacred Heart Hospital. hCECs were cultured as described previously [20,21]. Corneas were purchased from Eversight Eye Bank (Cleveland, OH, USA). Briefly, corneal endothelium, attached to the Descemet’s membrane, was stripped off, and incubated overnight at 37 °C in a humidified 5% CO_2_ chamber in growth media, composed of Opti-MEM-I (Invitrogen, Waltham, MA, USA) supplemented with 8% fetal bovine serum (FBS; Lonza, Walkersville, MD, USA), 5 ng/mL human recombinant EGF (Invitrogen), 20 ng/mL human recombinant nerve growth factor (Invitrogen), 100 μg/mL bovine pituitary extract (Invitrogen), 0.5 mM L-ascorbic acid 2-phosphate (Sigma-Aldrich Corp., St. Louis, MO, USA), 200 mg/L calcium chloride (Sigma-Aldrich Corp.), 0.08% chondroitin sulfate (Sigma-Aldrich Corp.), and 50 μg/mL gentamicin (Invitrogen). The next day, cells were trypsinized and seeded in 6-well plates coated with FNC coating Mix (Athena Enzyme Systems, Baltimore, MD, USA). Ages of donors were 25y, 32y, and 56y. hCECs were transfected with hsa-miR-302a (5′-UAAGUGCUUCCAUGUUUUGGUGA-3′ [22]; Bioneer Corp., Daejeon, Republic of Korea) or mimic negative control (miR-control, SMC-2002; Bioneer Corp.) using Lipofectamine™ RNAiMAX reagent (Invitrogen, Carlsbad, CA, USA) according to the manufacturer’s instructions. The cells were co-treated with IFN-γ (20 ng/mL; ab9659, Abcam, Cambridge, UK) for 48 h, after which the cells were collected for further experiments. 

### 2.2. Target Prediction and Pathway Analysis for miRNA-302a 

To understand the biological function and regulatory mechanism of hsa-miR-302a in hCECs, miRNA targets were predicted using miRBase and TargetscanHuman 8.0 (miRBase (https://www.mirbase.org/, (accessed on 12 November 2022) Manchester, UK) and TargetscanHuman 8.0 (https://www.targetscan.org/vert_80/, (accessed on 12 November 2022) Burlington, MA, USA) ) [23]. 

### 2.3. Cell Viability and Proliferation Assay 

hCECs (1 × 10^4^) were cultured in 96-well plates and treated with miRNA or IFN-γ for 48 h. Cell viability was measured using the Cell Counting kit-8 (CCK-8; Dojindo, Kumamoto, Japan). The plates were incubated with CCK-8 solution for 1–2 h. Cell viability was determined by measuring the absorbance at 450 nm using a microplate spectrophotometer (Synergy HTX, BioTek, Winooski, VT, USA). 

The cell proliferation rate was measured using a commercial bromodeoxyuridine (BrdU) proliferation assay kit (GmbH; Roche Diagnostics, Mannheim, Germany) according to the manufacturer’s protocol. Cells (5 × 10^3^ cells/well) were seeded into 96-well plates. Cells were labeled with BrdU at 37 °C and 5% carbon dioxide (CO_2_) for 48 h. After incubating the plate in the FixDent solution at 25 °C for 30 min, the cells were incubated with the anti-BrdU-POD solution at 25 °C for approximately 90 min. Then, the substrate solution was added to each well and the plate was incubated at 25 °C for 20 min. Thereafter, 1 M sulfuric acid (H_2_SO_4_) was added to each well to stop the reaction. Optical density was measured at 450 nm using a microplate reader (Synergy HTX, BioTek). Proliferation rates were expressed as the percentage of controls after subtraction of the corresponding blanks.

### 2.4. Cell Cycle Analysis 

Cell cycle analysis was performed using the Muse cell analyzer (Merck Millipore, Burlington, MA, USA) and propidium iodide (PI) staining according to the manufacturer’s protocol.

### 2.5. Immunofluorescent Staining and Autophagy Detection 

Samples were washed with phosphate-buffered saline (PBS) and fixed in 4% paraformaldehyde solution for 20 min. The cells were permeabilized for 10 min with 0.5% Triton X-100 and blocked with 1% bovine serum albumin (BSA) at 25 °C for 1 h. The cells were incubated overnight with either the rabbit anti-human nuclear factor kappa-light-chain-enhancer of activated B cells (NF-κB) antibody (sc-372; Santa Cruz Biotechnology, Santa Cruz, CA, USA), or the rabbit anti-X-box binding protein 1 (XBP-1) antibody (sc-7160; Santa Cruz Biotechnology), or the mouse anti-Ki67 antibody (sc-23900; Santa Cruz Biotechnology) at 4 °C, and then washed with PBS. The cells were incubated with either fluorescein isothiocyanate-conjugated goat anti-rabbit IgG antibody or anti-mouse IgG antibody (1:100) for 2 h at 25 °C in the dark, and then counterstained with Hoechst 33,342 nuclear staining dye (1:2000; Molecular Probes, Eugene, OR, USA) in accordance with the manufacturer’s instructions. The slides were observed under a fluorescence microscope (DMi8; Leica, Wetzlar, Germany) and photographs were taken.

CYTO-ID^®^ Autophagy Detection Kit (Enzo Life Sciences, Farmingdale, NY, USA) was used to measure autophagic vacuoles. Cells were incubated for 30 min in CYTO-ID indicators solution and then washed with PBS. The cells were observed under a fluorescence microscope (DMi8; Leica, Wetzlar, Germany) and photographs were taken. 

### 2.6. Enzyme-Linked Immunosorbent Assay

The conditioned medium was collected and frozen at −70 °C until the cytokine levels were determined. The levels of tumor necrosis factor-α (TNF-α) and interleukin-6 (IL-6) in the conditioned medium were assayed using commercial human TNF-α and IL-6 ELISA kits (R&D Systems, Minneapolis, MN, USA) according to the manufacturer’s protocols. In brief, the anti-human TNF-α and IL-6 antibodies were added to each well of a 96-well microtiter plate and incubated overnight at 25 °C. The next day, each well was washed with the washing buffer, filled with a blocking buffer containing 1% BSA, and then incubated at 25 °C for 1 h. One hundred microliters of experimental and standard samples were dispensed into the wells of a 96-well microtiter plate. The plates were sealed and incubated at 25 °C for 2 h. After incubation, the plates were washed four times, 100 μL of the detection antibody conjugated to horseradish peroxidase (HRP) was added to each well, and the plates were incubated at 25 °C for 2 h. One-hundred-microliter aliquots of a color reagent (3,3,5,5-tetramethylbenzidine [TMB]) were then applied to the plates for 20 min to obtain a blue color, and the reaction was stopped by adding 50 μL of 1 M H_2_SO_4_. The absorbance was measured at 450 nm using an automatic plate reader (Synergy HTX, BioTek, Winooski, VT, USA) with a reference wavelength of 570 nm.

### 2.7. Western Blotting

Radioimmunoprecipitation assay buffer (Biosesang, Seoul, Republic of Korea) containing protease (11836153001, Complete Mini Protease Inhibitor Cocktail Tablets; Roche, Basel, Switzerland) and phosphatase inhibitor cocktails (04906837001, PhosSTOP; Roche) was used to isolate the total cellular proteins. Western blotting was performed using standard protocols. A 5% skim milk was used for blocking the non-specific binding for 1 h. Primary antibodies included the anti-ERK1/2 (sc-514302; 1∶500 dilution; Santa Cruz, CA, USA), rabbit anti-pERK1/2 (ab214362; 1∶500 dilution; Santa Cruz), mouse anti-microtubule-associated protein 1A/1B-light chain 3 (LC3, M186-3; 1∶1000 dilution; MBL), mouse anti-activating transcription factor 6 (ATF6, PA5-20215; 1∶500 dilution; Santa Cruz), or rabbit anti-GAPDH (LF-PA0212; 1∶5000 dilution; Abfrontier) antibodies. HRP-conjugated secondary antibody and a WEST-Queen™ Western Blot Detection Kit (iNtRON Biotechnology, Seongnam, Republic of Korea) were used to detect the immunoreactive bands. The protein bands were quantified using the ImageJ Gel Analysis program.

### 2.8. SA-β-gal Assay and Cell Size Measurement

SA-β-gal staining was performed using a senescence-β-galactosidase staining kit (Biovision, Milpitas, CA, USA). Briefly, after removing the growth media from the cells, the cells were rinsed once with PBS. A fixative solution was added to each well. The cells were fixed for 10–15 min at 25 °C. After washing the cells with PBS, the cells were incubated in β-galactosidase staining solution at 37 °C overnight in a dry incubator.

Cell size was measured using AxioVision 4.8 (Carl Zeiss, Oberkochen, Germany). After pictures of cells were taken, cell borders were outlined, and the area of the outlined region was automatically calculated using AxioVision software.

### 2.9. Mitochondrial Oxidative Stress Evaluation and MitoPotential Assay

Mitochondrial superoxide production was measured using MitoSOXTM Red (M36008, Invitrogen), according to the manufacturer’s protocol. The cells were incubated with 5 μM MitoSOXTM reagent at 37 °C in the dark for 10 min. Fluorescence intensity in each well was measured using a Cytoflex analyzer (Beckman Coulter Life Sciences, Miami, FL, USA) and the intensity of the MitoSOXTM red fluorescence was calculated.

The Muse™ MitoPotential assay (Merck Millipore, Burlington, MA, USA) was used to evaluate the mitochondrial membrane potential. The assay utilizes the MitoPotential dye, a cationic lipophilic dye, to detect changes in the mitochondrial membrane potential, and 7-amino-actinomycin D (7-AAD) as an indicator of cell death. The data were analyzed using the Muse™ Cell Analyzer (Merck Millipore). 

### 2.10. Real Time Reverse Transcription Polymerase Chain Reaction (RT-qPCR) 

Real time reverse transcription polymerase chain reaction was performed for interleukin-8 (IL-8) and C-C motif chemokine ligand 2 (CCL2). The ReliaPrep™ RNA Miniprep Systems (Promega Cooperation, Madison, WI, USA) was used to extract total RNA. The Nanodrop method was used to measure RNA concentrations. The GoScript™ Reverse Transcription System (Promega Cooperation) was used to synthesize first-strand cDNA from 0.5 μg of total RNA with oligonucleotide primers. AccuPower^®^ 2X GreenStar™ qPCR Master Mix (Bioneer) with RT-qPCR primer was used to perform RT-qPCR. The thermocycling parameters were as follows: 95 °C for 10 min, 40 cycles at 95 °C for 15 s, and at 60 °C for 1 min [24]. SYBR green fluorescence intensity was taken. The *GAPDH* gene served as a refence gene. Melting curve analysis was employed to identify the purity of amplified products. The ΔΔCq method was used to analyze RT-qPCR [24]. The reverse transcription primers were described in Table S1.

### 2.11. In Vivo Transfection and Evaluation of Corneal Endothelium in Rat

This study was approved by the Institutional Animal Care and Use Committee of Hallym University Medical Center (HMC 2021-3-1007-38). All procedures were performed according to the Association for Research in Vision and Ophthalmology Statement for the Use of Animals in Ophthalmic and Vision Research. Six-week-old Sprague-Dawley (SD) rats (Raonbio, Yong-in, Republic of Korea) were used for this procedure. Four SD rats were included in each group. Rats were maintained in a colony room with a 12/12-h light/dark cycle at 25 °C for 7 days before beginning the experiments. 

MicroRNAs were transfected into the corneal endothelium of rats by electroporation. microRNAs (0.5 nmole) were injected into the anterior chamber of rats, and electroporation was performed using 7 mm Tweezer and the ECM830 electroporation system (BTX Havard apparatus, Holliston, MA, USA; setting was 100 mV, 100 ms duration, 950 ms interval, and 5 pulses). Then, the corneas were cryoinjured for 15 s by 3 mm diameter metal rods chilled with liquid nitrogen. The clinical evaluation, including corneal opacity and photography, was performed on day 1, day 4, and day 7. Corneal opacity was graded as follows: grade 0 = clear cornea, grade 1 = mild corneal opacity and still allowing good visibility of details of the iris, grade 2 = moderate corneal opacity with partial masking of the iris, and grade 3 = severe corneal opacity without view of the iris. 

For alizarin S red staining, a solution of 0.2% alizarin red S (Sigma-Aldrich, St. Louis, MO, USA) in 0.9% NaCl (pH 4.2) was injected into the anterior chamber for 90 s. The corneas were then fixed in 2.5% glutaraldehyde (Sigma-Aldrich). Then, the corneal buttons were obtained, and mounted with a drop of 0.9% NaCl. The corneal endothelium was observed under the microscopy (Leica DMi8; Leica, Wetzlar, Germany), and photographs of the central endothelium were taken (Las X, Leica, Wetzlar, Germany). Cells were counted manually at × 400 magnification. 

Immunohistochemistry of IFN-γ was performed. Samples were fixed in 4% paraformaldehyde solution and embedded in paraffin. Sections of 5 μm thick were used for histological staining. The tissue sections were hydrated, and antigen retrieval was performed using a microwave oven in citrate buffer (pH 6.0). The slides were blocked with 1% bovine serum albumin (BSA) at 25 °C for 1 h and incubated overnight with the mouse anti- IFN- γ antibody (sc373727, Santa Cruz Biotechnology) at 4 °C, and then washed with PBS. Secondary biotinylated antibody was applied for 2 h at 25 °C. Vectastain Universal Elite ABC staining kit (Vector Laboratories, Burlingame, CA, USA) and 3, 3–diaminobenzidine peroxidase substrate (Vector Laboratories) were used for immunodetection. Hematoxylin staining was performed as nuclear counterstaining. The slides were observed using light microscopy (DMi8; Leica, Wetzlar, Germany). 

### 2.12. In Vivo Staining of MitoTracker Green FM Fluorescent Probe and MitoSOX Probe

To evaluate mitochondrial mass and oxidative stress, MitoTracker green FM fluorescent probe (M7514, Invitrogen) and MitoSOX probe (M36008, Invitrogen) were used for staining the corneal endothelium. Briefly, the corneas were excised from the eyeball and incubated in 200 nM MitoTracker green reagent and 5 µM MitoSOX reagent for 10 min at 37 °C in the dark. After washing with PBS, the corneas were flat-mounted and observed under a microscope (Leica DMi8, Leica Microsystems, Wetzlar, Germany).

### 2.13. Statistics

Data are expressed as the mean ± standard deviation. Statistical analyses were performed using unpaired Student’s *t*-test for two-group comparisons and one-way analysis of variance (ANOVA), followed by Tukey’s multiple comparison test for more than two groups using GraphPad Prism v.9 (GraphPad Software, San Diego, CA, USA). All experiments were repeated at least three times. 

## 3. Results

### 3.1. MiR-302a Promotes the Proliferation of hCECs

**Figure 1:**
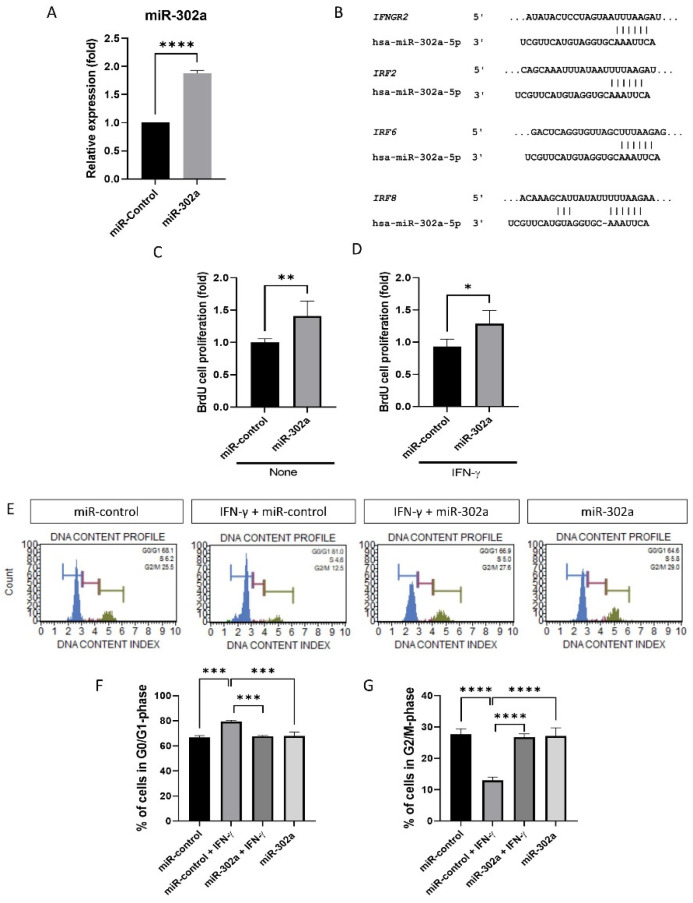
Effect of miR-302a on cell proliferation. (**A**) Expression of miR-302a was elevated. (**B**) Predicted miR-302a–binding sites in the 3′-UTR of the *IFNGR2*, *IRF2*, *IRF6,* and *IRF8* mRNA indicate the interaction between hsa-miR-302a and IFN-γ signaling pathway. Perfect matches in seed regions are indicated by lines. miRNA targets were predicted using miRBase and TargetscanHuman 8.0. (**C**,**D**) BrdU incorporation assay was performed in miR-control and miR-302a with or without interferon-γ (IFN-γ). (**E**) Cell cycle analysis was performed, and cell cycle graph was shown. (**F**,**G**) IFN-γ induced the cell cycle arrest, which was eliminated by miR-302a. * *p* < 0.05, ** *p* < 0.01, *** *p* < 0.001 and **** *p* < 0.0001.

Expression of miR-302a was elevated (Figure 1A). miR-302a transfection amplified cell proliferation, with or without IFN-γ treatment (Figure 1C,D). Predicted miR-302a–binding sites in the 3′-UTR of the *IFNGR2, IRF2, IRF6,* and *IRF8* mRNA indicate the interaction between hsa-miR-302a and the IFN-γ signaling pathway (Figure 1B). Perfect matches in seed regions are indicated by lines. miR-302a transfection amplified the cell proliferation, with or without IFN-γ treatment (Figure 1C,D). The cell cycle was shifted from the G0/G1 to S and G2/M phases (Figure 1E). The proportion of G0/G1-phase cells was elevated after IFN-γ treatment compared to the miR-control, miR-302a+IFN-γ, and miR-302a cells (Figure 1F), while those in the S-phase or G2/M phase were reduced with IFN-γ treatment compared with the miR-control, miR-302a+IFN-γ, and miR-302a cells (Figure 1G).

### 3.2. MiR-302a Suppresses Oxidative Stress and Senescence

**Figure 2:**
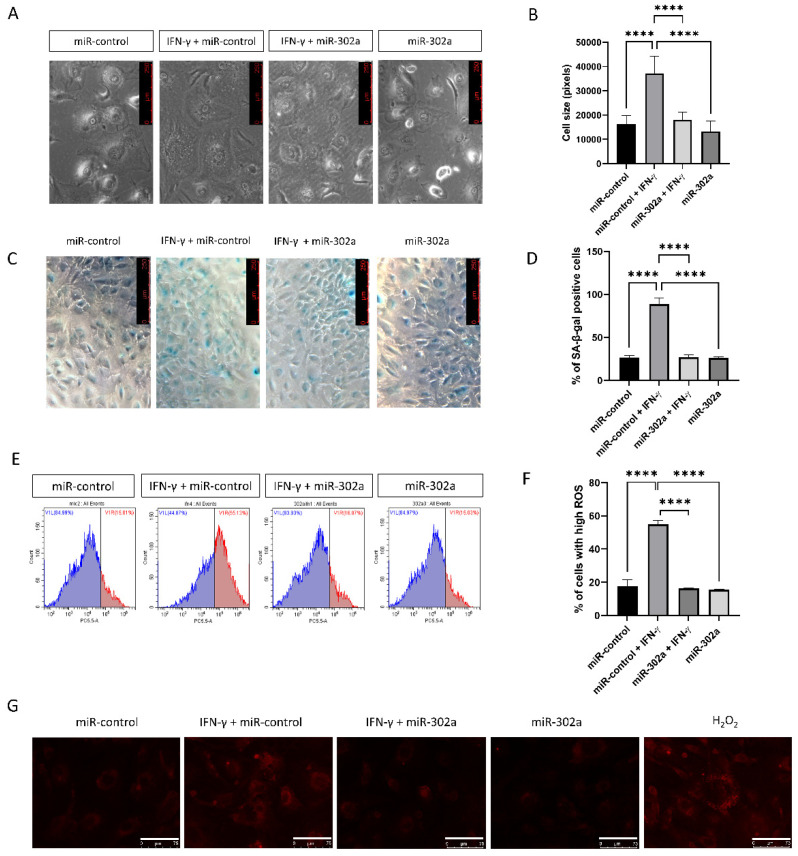
Effect of miR-302a on senescence. (**A**,**B**) Cell size was evaluated in miR-control and miR-302a with or without interferon-γ (IFN-γ). Scale bar = 250 µm. (**C**,**D**) Senescence-β-galactosidase staining was performed and blue staining indicated the senescence-β-galactosidase staining positivity. (**E**–**G**) Mitochondrial oxidative stress levels were measured using MitoSOX probe. Scale bar = 75 µm. Data presented as mean ± S.D. **** *p* < 0.0001.

To assess cellular senescence in vitro, cell size was evaluated because senescent cells are characterized by enlarged and flattened cell morphology. IFN-γ treatment promoted the enlargement of hCECs, which were eliminated by miR-302a (Figure 2A,B). We performed SA-β-gal assay, which is a marker of senescence. The SA-β-gal assay revealed an increase in the number of senescent cells with IFN-γ treatment, which was eliminated by miR-302a (Figure 2C,D). We used MitoSOX probe to evaluate mitochondrial oxidative stress levels as a property associated with senescence. IFN-γ treatment increased mitochondrial oxidative stress levels, which were eliminated by miR-302a (Figure 2E–G). 

### 3.3. MiR-302a Suppresses Senescence-Induced Inflammation

**Figure 3:**
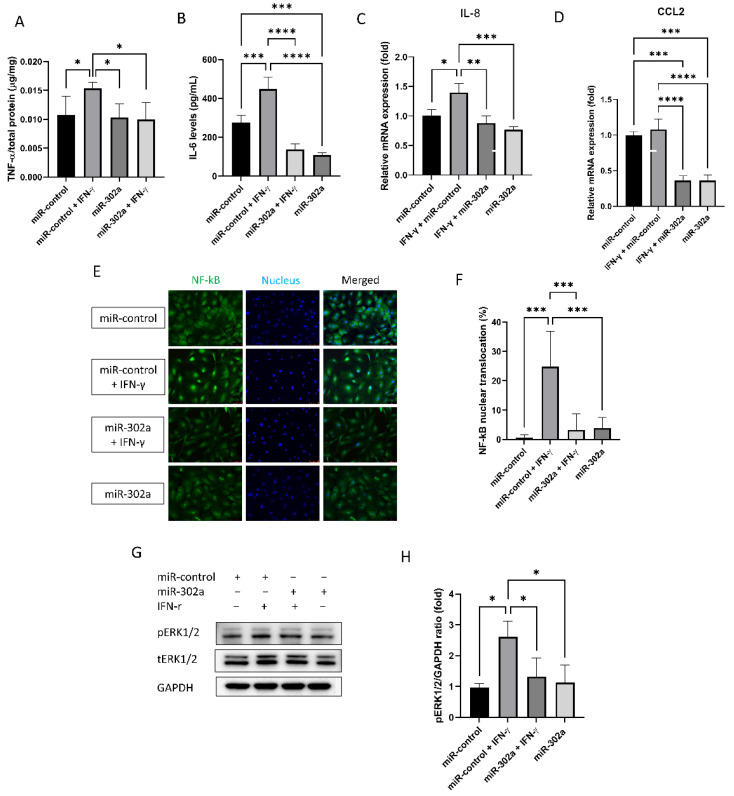
Effect of miR-302a on senescence-associated response. Senescence-associated secretary phenotype secretion (**A**,**B**) was measured in conditioned medium. (**C**,**D**) mRNA expression of *IL-8* and *CCL2* was evaluated by real-time PCR. (**E**,**F**) Nuclear factor-κB (NF-κB) translocation was evaluated using immunofluorescence staining. Scale bar = 75 µm. (**G**,**H**) Activation of extracellular-signal-regulated kinase (ERK) 1/2 was evaluated with or without IFN-γ or miR-302a. Data presented as mean ± S.D. * *p* < 0.05, ** *p* < 0.01, *** *p* < 0.001 and **** *p* < 0.0001.

Senescent cells secrete the senescence-associated secretory phenotype (SASP) factors. The secretion of TNF-α and IL-6, known as the SASP factors, was amplified by IFN-γ treatment, which was eliminated by miR-302a (Figure 3A,B). mRNA expression of *IL-8* and *CCL2* was evaluated by real-time PCR (Figure 3C,D). IFN-γ treatment elevated mRNA expression of *IL-8* and *CCL2*, which was eliminated by miR-302a. We performed immunofluorescence staining of NF-κB and Western blot analyses of ERK1/2 to investigate the signaling pathways of secretion SASP factors. Nuclear translocation of NF-κB, which is an inflammatory signaling pathway, was promoted by IFN-γ treatment, which was eliminated by miR-302a (Figure 3E,F). Phosphorylation of ERK1/2, which indicates the activation of ERK1/2 signaling pathway, was enhanced by IFN-γ treatment, which was eliminated by miR-302a (Figure 3G,H). 

### 3.4. MiR-302a Suppresses IFN-γ-Induced Cell Death

**Figure 4:**
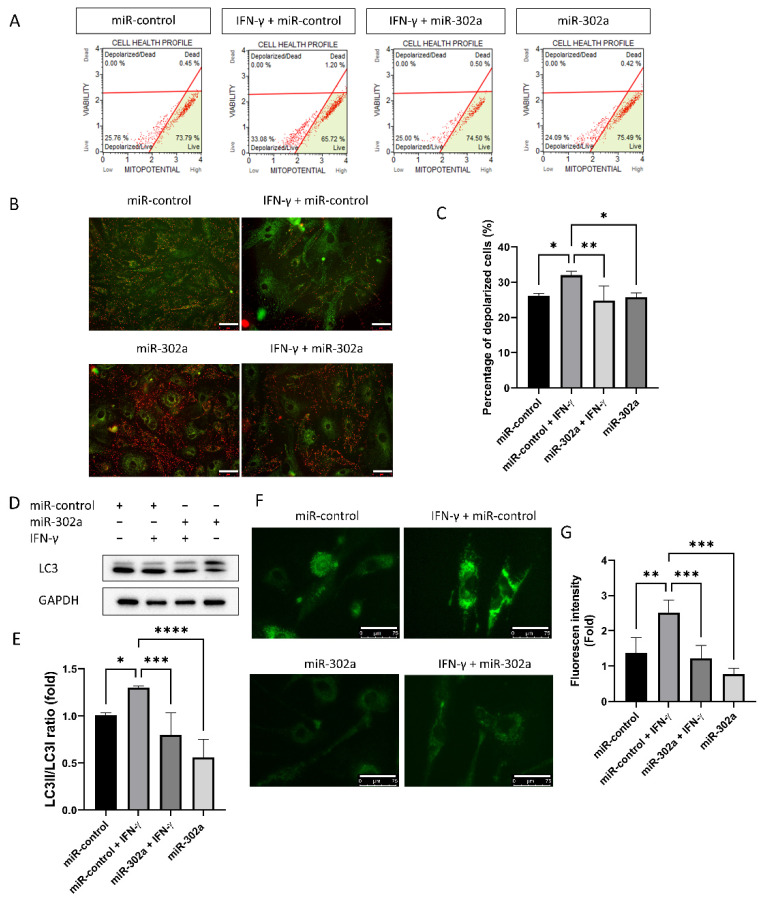
Effect of miR-302a on IFN-γ-induced cell death. (**A**–**C**) Mitochondrial membrane potential was measured by cell analyzer using Mitopotential kit, and by fluorescence imaging using JC-1 with or without IFN-γ or miR-302a. Scale bar = 75 µm. (**D**,**E**) Autophagy was measured by the conversion of LC3I to LC3II. (**F**) Autophagosome was stained using autophagy detection kit. Scale bar = 75 µm. (**G**) Fluorescence intensity of autophagosome was measured using autophagy detection kit. Data presented as mean ± S.D. * *p* < 0.05, ** *p* < 0.01, *** *p* < 0.001 and **** *p* < 0.0001.

The Muse mitopotential assay and JC-I probe were used to assess mitochondrial membrane potential as a mitochondrial function. The mitochondrial membrane potential measured by Muse mitopotential kit was depolarized by IFN-γ treatment, which was eliminated by miR-302a (Figure 4A,B). The mitochondrial depolarization is indicated by a reduction in the red to green fluorescence intensity ratio of JC-1. The red fluorescence intensity of JC-1 was reduced by IFN-γ treatment, which was eliminated by miR-302a (Figure 4C). To investigate autophagy, Western blot analyses for LC3 were performed. LC3I was converted to LC3II by IFN-γ treatment, which was eliminated by miR-302a (Figure 4D,E). Autophagic vacuoles were evaluated using the CYTO-ID^®^ Autophagy Detection Kit (Figure 4F). Prominent autophagic vacuoles were observed in IFN-γ treated cells, which were reduced by miR-302a. 

### 3.5. MiR-302a Suppresses Endoplasmic Reticulum Stress

**Figure 5:**
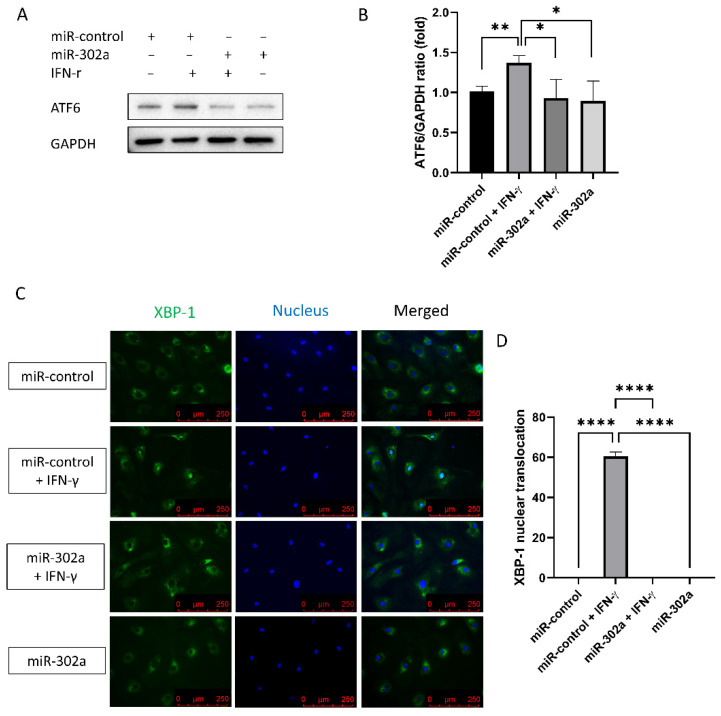
Effect of miR-302a on IFN-γ-induced ER stress. (**A**,**B**) ATF6 levels were elevated by IFN-γ treatment, which was eliminated by miR-302a. (**C**,**D**) Nuclear translocation of XBP-1 was evaluated in control and miR-302a group. Data presented as mean ± S.D. * *p* < 0.05, ** *p* < 0.01 and **** *p* < 0.0001.

To assess ER stress levels, Western analysis of ATF6 levels and immunofluorescence staining of XBP-1 were performed. ATF6 levels, which are a sensor of ER stress, were elevated by IFN-γ treatment, which was eliminated by miR-302a (Figure 5A,B). Nuclear translocation of XBP-1, which indicates the activation of ER stress, was amplified by IFN-γ treatment, which was eliminated by miR-302a (Figure 5C,D).

### 3.6. In Vivo Transfection and Evaluation of miR-302a Regenerates Corneal Endothelium in Rat

**Figure 6:**
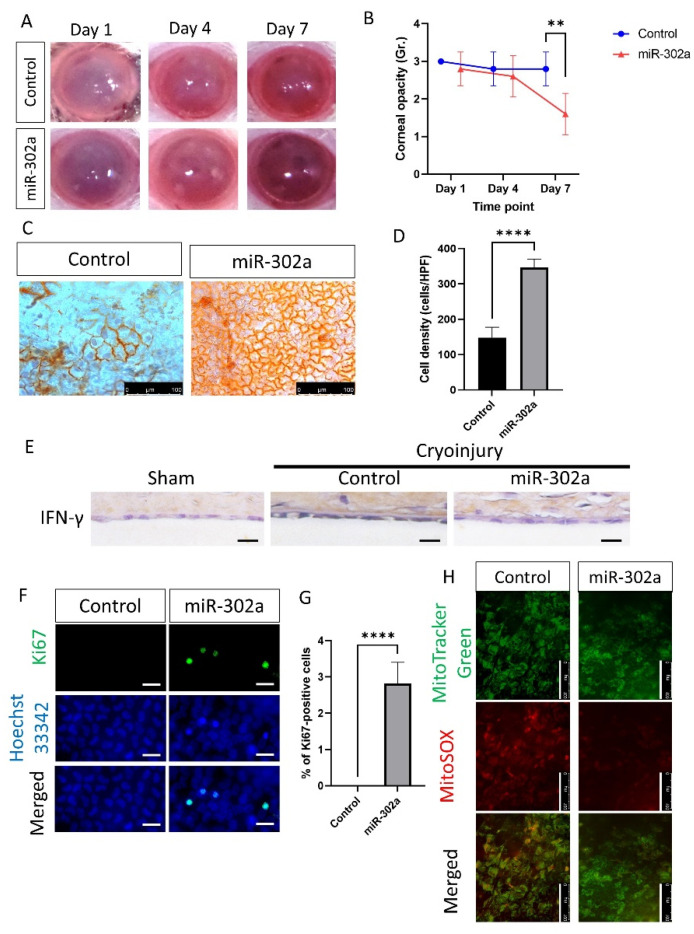
In vivo transfection of miR-302a and evaluation of corneal endothelium in rat. (**A**,**B**) Corneal opacity was evaluated in control and miR-302a group on day 7. (**C**,**D**) Alizarin S red staining showing corneal endothelium in control and miR-302a group on day 7. (**E**) Immunohistochemistry of IFN-γ was performed on the rat corneal endothelium. Scale bar = 25 µm. (**F**,**G**) Immunofluorescence staining of Ki67 was performed in corneal endothelium of rats. Scale bar = 25 µm. (**H**) MitoTracker green and MitoSOX staining was performed in corneal endothelium of rats in control and miR-302a group. Scale bar = 100 µm. Data presented as mean ± S.D. ** *p* < 0.01 and **** *p* < 0.0001.

Corneal opacity, which indicates CEC function, was lower in the miR-302a group compared to the control at day 7 (*p* = 0.005; Figure 6A,B). Alizarin S red staining showed that the number of CECs was higher in the miR-302a group compared to the control at day 7 (*p* < 0.001, Figure 6C,D), suggesting the regeneration in the miR-302a group. Immunohistochemistry of IFN-γ was performed to evaluate whether cryoinjury increased IFN-γ levels in the rat corneal endothelium. Cryo-injured corneal endothelium showed an increase in IFN-γ, which was eliminated by miR-302a (Figure 6E). 

To investigate the proliferation of CECs, immunofluorescence staining of Ki67, a proliferation marker, was performed. The number of Ki67-positive cells was higher in the miR-302a group compared to the control (*p* < 0.001; Figure 6F,G), suggesting the higher proliferation rate of CECs in the miR-302a group. To assess mitochondria states, MitoTracker green for mitochondrial shape and MitoSOX red for mitochondrial oxidative stress level were employed. MitoTracker green and MitoSOX staining of rat CECs showed a lower level of mitochondrial oxidative stress in the miR-302a group, compared to the control group (Figure 6H).

## 4. Discussion

In this study, we found that miR-302a regulates the regeneration and prevents IFN-γ-induced cell death of cultured hCECs. miR-302a elevated the cell proliferation in hCECs and shifted the cell cycle from the G0/G1 phase to the S and G2/M phases. miR-302a directly or indirectly targets cell cycle regulators including p21, p53, large tumor suppressor kinase 2 (LATS2), phosphatase and tensin homolog (PTEN), and cyclin E [25,26]; their expression in primary and transformed cell lines enhances an increase in S-phase and a decrease in G1-phase cells [9]. The miR-302/367 cluster is a direct target of GATA binding protein 6 (GATA6) [27,28], which is a zinc finger transcription factor required for differentiation, and regulates progenitor proliferation and differentiation, as well as apical-basal polarity [29]. Sex determining region Y-box 2 (SOX2), which is a transcriptional factor maintaining the stem cell properties and promoting proliferation [30,31], binds to the conserved promoter region of miR-302 and regulates the expression of miR-302a [9]. miR-302a accelerates mesenchymal-to-epithelial changes [32], which means reprogramming features that activate stem cells [33,34].

Senescence is thought to be one of the most important mechanisms to prevent the regeneration of hCECs [20]. In this study, IFN-γ induced senescence, which is characterized by an increase in cell size, SA-β-gal activity, and mitochondrial oxidative stress level, but its effect was reduced by miR-302a [35]. p21, p27, and p53, which mediate senescence, are upregulated by IFN-γ [36]. miR-302a inhibits interferon regulatory factor-5 and associated cytokine secretion [14], and prevents the up-regulation of p21 and p27 [37]. p53, which regulates mitochondrial function and promotes senescence [38], is directly suppressed by miR-302a [39]. In this study, IFN-γ increased the secretion of SASP and caused the nuclear translocation of NF-κB and the change in ERK1/2 activation, but the effects were eliminated by miR-302a. Senescent cells are the source of inflammation due to secreting SASP factors such as IL-6, IL-7, IL-8, CCL2, and TNF-α [40,41]. IFN-γ, one of the SASP factors, activates NF-κB which moves to the nucleus and induces the expression of pro-inflammatory genes [42]. NF-κB activation also leads to an increase in reactive oxygen species (ROS) levels via the induction of nicotinamide adenine dinucleotide phosphate (NADPH) oxidase 4 (NOX4), which promotes SASP production and senescence via p16 [42]. IFN-γ activates the p38/ERK1/2 signaling pathways, resulting in epithelial–mesenchymal transition, and inflammation [43,44]. ERK1/2 activation is accompanied by cellular senescence and promotes inflammation [45,46]. miR-302a has been reported to inhibit the mitogen-activated protein kinase/ERK1/2 pathway [47,48], and to negatively regulate the signal transducer and activator of the transcription 1 (STAT1)/NF-κB pathway [49]. In addition, miR-302 has a protective effect against oxidative stress by targeting *CCL5,* and it also regulates human fibroblast proliferation [50,51]. 

IFN-γ regulates cell death, including necroptosis, apoptosis, and autophagy [52,53]. In CECs, IFN-γ induces inflammasome-mediated death of CECs [18]. In this study, miR-302a protected against IFN-γ-induced depolarization of the mitochondrial membrane potential. Mitochondrial membrane potential maintenance is important for cell function and death [54], because it is necessary for energy production, and its loss is essential for DNA damage and growth factor deprivation [49,55]. Depolarization of mitochondrial membrane potential is associated with the opening of the mitochondrial permeability transition pore, which occurs early in the apoptosis process [56]. Apoptosis is initiated by cytochrome c release from the mitochondria [57]. IFN-γ impairs energy expenditure and metabolism in mitochondria by suppressing complex II and SIRT1, and by activating RNase L [58,59]. Mitochondrial injury by IFN-γ causes apoptosis through loss of mitochondrial membrane potential and accumulation of ER stress [54,59,60]. miR-302 translocated into the mitochondria by forming a complex with Ago2 [61,62] and affected mitochondrial dynamics through the C-C motif ligand 5 [51]. In this study, miR-302a suppressed IFN-γ-induced autophagy, which was evaluated by the conversion of LC3I to LC3II and IFN-γ-induced ER stress. This was further evaluated by ATF6 and XBP1 [63,64,65]. ER stress, which is induced by IFN-γ, and it triggers apoptosis, stimulates autophagy, and switches autophagy to cell death [66]. IFN-γ induces ER stress by the cleavage of caspase-4 and activation of protein kinase RNA-like endoplasmic reticulum kinase (PERK) and inositol-requiring-1α (IRE1α) pathways [60,64]. The ER stress senor system consists of IRE-1α, PERK, and ATF6 [67]. XBP1, a downstream of IRE1α, moves into the nucleus and initiates the ER stress response genes linking to inflammation [68,69]. ATF6 increases expression of TNF-α and other inflammatory cytokines [70,71]. The ATF6 branch of the ER stress response is implicated by calreticulin, a Ca^2+^- dependent ER chaperone [72]. Depletion of calreticulin and Ca^2+^ stores in the ER is associated with a decrease in miR-302 [73]. In this study, we showed that miR-302a ameliorates cell death by regulating mitochondria/ER stress. Further study is necessary to evaluate the inhibitor for miR-302a, despite the effect of the miR-302a-5p inhibitor on cytokine mRNA expression in hCECs. The miR-302a-5p inhibitor elevated cytokines including IL-6, IL-8, TNF-α, and CCL2 (Figure S1). 

**Figure 7:**
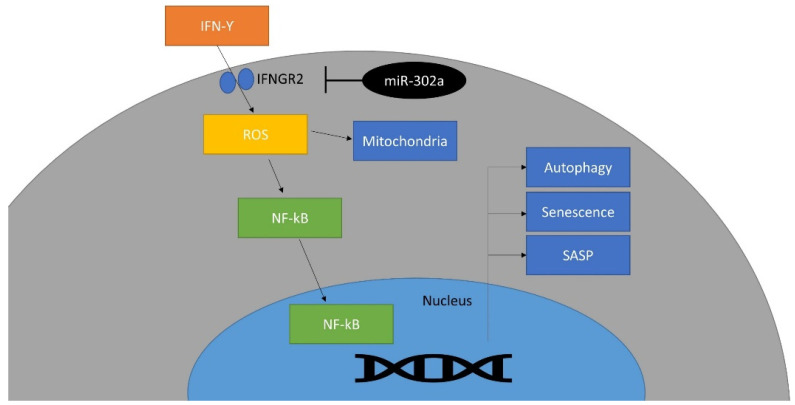
Schematic diagram of the effect of miR-302a on IFN-γ signaling pathway. This miRNA targets *IFNGR2* and inhibits IFN-γ signaling pathway.

In vivo study showed that miR-302a protects the CECs against cryoinjury and promotes the regeneration of CECs, which improves the corneal opacity. This is compatible with in vitro experiments. Cryoinjury induced the activation of the inflammatory response, as well as the damage to rat CECs. In this study, cryoinjury induced an increase in IFN-γ in the rat corneal endothelium. IFN-γ has been reported to increase in CEC diseases and to induce CEC death through inflammasomes [17,18]. miR-302a increased expression of Ki67, which is a proliferation marker and increases during regeneration [74,75]. Regeneration is regulated by mitochondria because energy production in mitochondria is essential for proliferation [76]. Mitochondrial function is reversely correlated with oxidative stress level although mitochondria are the major source of ROS [77]. Oxidative stress activates p53, a senescence-associated protein, and inhibits proliferation [78]. Reduced oxidative stress level restores mitochondrial function, promotes ATP production and proliferation [79]. Mitochondrial oxidative stress levels were reduced by miR-302a in the in vivo study. miR-302a regulates the biological signaling against oxidative stress [50,80] and controls the cell cycle [81]. In vivo gene therapy, using miR-302a in CECs, may be used as a treatment for remaining CECs, through the regeneration of CECs. Alternatively, the development of a drug that activates miR-302a may be helpful. In addition, it should be possible to transplant CECs by proliferating cells in vitro using miR-302a in CECs. Further studies for safety are required before clinical use. A schematic diagram is shown in Figure 7.

In conclusion, miR-302a enhanced the regeneration of hCECs by eliminating IFN-γ-induced senescence and ER stress, and promoted the CECs regeneration in rats after cryo-injury. Therefore, miR-302a may be a therapeutic option for the protection and regeneration of hCECs against pathological stress. 

## Data Availability

All the data utilized in this study are available upon request to the corresponding author.

## Supplementary Materials

The following supporting information can be downloaded at: https://www.mdpi.com/article/10.3390/cells12010036/s1, Figure S1: Effect of miR-302a-5p inhibitor on cytokine mRNA expression. Table S1. Primers for RT-PCR.
