# Supplementary material for: MiR-302a Regenerates Human Corneal Endothelial Cells against IFN-γ-Induced Cell Death
Source: Cells. 2022 Dec 22;12(1):36. doi: 10.3390/cells12010036 (PMC9818234; doi:10.3390/cells12010036)

Table S1. Primers for RT-PCR

Gene	Primer	Primer sequence (5′ to 3′)
IL-8	Forward	GAGAGTGATTGAGAGTGGACCAC
	Reverse	CACAACCCTCTGCACCCAGTTT
CCL2	Forward	AGAATCACCAGCAGCAAGTGTCC
	Reverse	TCCTGAACCCACTTCTGCTTGG
miR-302a	Forward	CTAGCCTGCAGGTCGAAGAGTAAGTGCTTCCATG
	Reverse	ATCCGGCCGGCCGTGCAATTCCAATTTCTATTCA
GAPDH	Forward	CTGGGCTACACTGAGCACC
	Reverse	AAGTGGTTGAGGGCAATG

Figure S1. Effect of miR-302a-5p inhibitor on cytokine mRNA expression. (A-D) mRNA expressions of IL-6, IL-8, TNF-α and CCL2 increased by miR-302a-5p inhibitor.

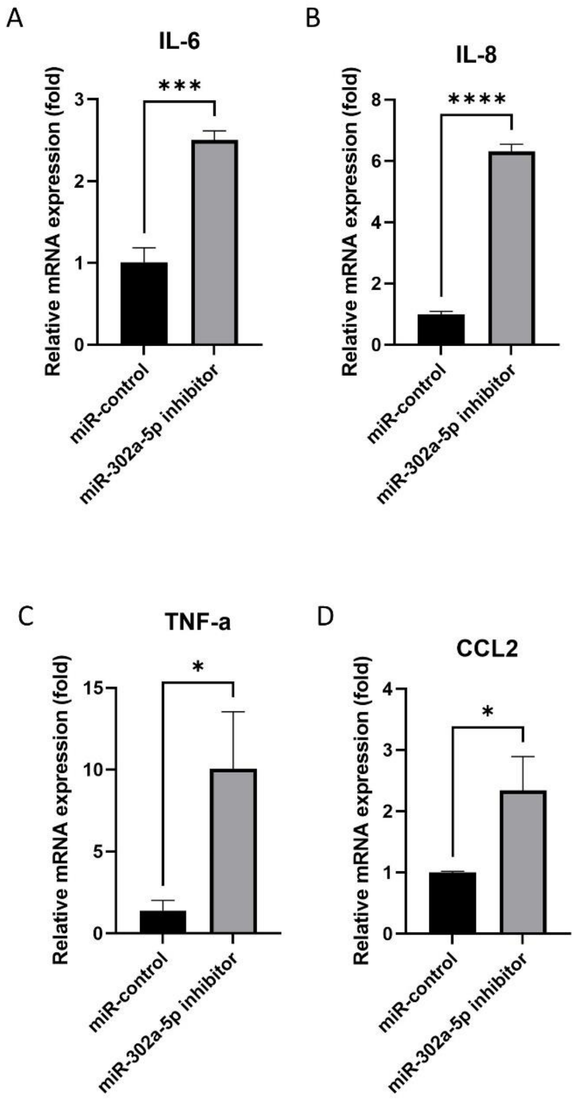

Supplement: Supplementary file 1 [file cells-12-00036-s001.zip › cells-2061839-supplementary.pdf]
